# Orally disintegrating tablets containing famotidine nanoparticles provide high intestinal absorbability *via* the energy-dependent endocytosis pathway

**DOI:** 10.3389/fbioe.2023.1167291

**Published:** 2023-03-10

**Authors:** Noriaki Nagai, Fumihiko Ogata, Reita Kadowaki, Saori Deguchi, Hiroko Otake, Yosuke Nakazawa, Mayumi Nagata, Hiroshi Sasaki, Naohito Kawasaki

**Affiliations:** ^1^ Faculty of Pharmacy, Kindai University, Higashi-Osaka, Japan; ^2^ Faculty of Pharmacy, Keio University, Minato-ku, Japan; ^3^ Department of Ophthalmology, Dokkyo Medical University, Tochigi, Japan; ^4^ Department of Ophthalmology, Kanazawa Medical University, Shimotsuga-gun, Japan

**Keywords:** famotidine, nanoparticle, orally disintegrating tablet, endocytosis, intestinal absorption, freeze-drying

## Abstract

The permeability of the Biopharmaceutics Classification System (BCS) class III drugs are low, and their oral bioavailability needs to be improved. In this study, we attempted to design oral formulations containing famotidine (FAM) nanoparticles to overcome the limitations of BCS class III drugs. Dispersions containing FAM nanoparticles with a particle size of approximately 50–220 nm were produced by the bead-milling treatment. Moreover, we succeeded in preparing an orally disintegrating tablet containing FAM nanoparticles using the dispersions described above, additives (D-mannitol, polyvinylpyrrolidone, and gum arabic), and freeze-dry treatment (FAM-NP tablet). The FAM-NP tablet was disaggregated 3.5 s after addition to purified water, and the FAM particles in the redispersion of the FAM-NP tablet stored for 3 months were nano-sized (141 ± 6.6 nm). The *ex-vivo* intestinal penetration and *in vivo* absorption of FAM in rats applied with the FAM-NP tablet were significantly higher than those in rats applied with the FAM tablet containing microparticles. In addition, enhanced intestinal penetration of the FAM-NP tablet was attenuated by an inhibitor of clathrin-mediated endocytosis. In conclusion, the orally disintegrating tablet containing FAM nanoparticles improved low mucosal permeability and low oral bioavailability and overcame these issues of BCS class III drugs as oral formulations.

## 1 Introduction

Gastric and duodenal ulcers, also known as peptic ulcers, are caused by damage to the gastrointestinal mucosa induced by pepsin and acid and are the most common of gastrointestinal diseases (Malfertheiner et al., 2009). The control of acid secretion has been used as a key therapeutic target for ulcer diseases in clinical settings. Acid secretion can be reduced by the inhibition of the histamine type 2 (H_2_) receptor, since histamine is a major stimulant of acid secretion through H_2_ receptors. Thus, H_2_ receptor antagonists, which block H_2_ receptors reversibly and competitively, are useful for the treatment of peptic ulcers (Huang and Hunt, 2001). Famotidine (FAM) is a competitive inhibitor of H_2_ receptors, and it was reported that the oral FAM (40 mg daily) is well tolerated, and there have been no serious adverse experiences attributed to its administration (Lyon, 1986; Ramchandran et al., 2011). The inhibitory effect of FAM was reported to be 20- and 7.5-fold higher than that of cimetidine and ranitidine, respectively (Hassan et al., 1990; Patel and Patel, 2013).

Oral administration is the preferred route for drug delivery, and many drugs, including FAM, are administered orally because they provide higher patient compliance. However, FAM has a low mucosal permeability, and is classified as class III in the Biopharmaceutics Classification System (BCS) classification, which is used to determine the rate-limiting factors of the gastrointestinal absorption process of various drugs (Khalifa and Abdul Rasool, 2017). Drugs of this class (BCS class III) exhibit poor and variable bioavailability (BA) *via* low intestinal penetration. Specifically, the intestinal penetration and absolute BA of FAM after oral administration are less of 50% and 40%–45%, respectively (Jha et al., 2014). Thus, FAM is incompletely absorbed from the gastrointestinal tract and a large proportion of the drug is eliminated from the alimentary canal before absorption (Mady et al., 2010; Jha et al., 2014). Therefore, developing an FAM formulation with unique properties is important for overcoming these issues.

Gastric emptying, intestinal permeability, and dissolution rate are related to the oral BA of a drug (Hassan et al., 1990; Patel and Patel, 2013), thus the enhancement of dissolution rate and solubility is effective in improving the low BA. During the past few decades, many approaches have been developed to enhance its intestinal permeability, dissolution rate and solubility, such as co-solvents, salts, amorphous molecules, liquisolid formulation, nanoparticles, complexation with cyclodextrin, liposomes, and micelles (Hassan et al., 1990; Fahmy and Kassem, 2008; Müllertz et al., 2010; Matsuda et al., 2011; Williams et al., 2013; Kumar et al., 2014; Jog and Burgess, 2017; Khalifa and Abdul Rasool, 2017). In past studies, we also reported that preparation of the drug in nanocrystalline dispersions increases the dissolution rate, solubility, and intestinal penetration *via* energy-dependent endocytosis (Nagai et al., 2020; Deguchi et al., 2021; Nagai et al., 2022). Moreover, we prepared the oral formulation based on irbesartan nanocrystals, BCS class II (low solubility and high permeability), and showed the formulation improve the drug solubility and absorbability (Nagai et al., 2022). However, nanocrystalline dispersions are chemically and physically unstable (Abdelwahed et al., 2006), and the oral formulation based on irbesartan nanocrystals for more than 1 month has not been confirmed, nor is it known whether this formulation for preparing tablet is applicable to drugs other than BCS Class II. Therefore, the further study for production of dried powders and tablets containing nanocrystalline dispersions is important for their practical application in oral formulations (Kumar and Burgess, 2014; Liu et al., 2014). In this study, we attempted to design molded tablets containing FAM nanoparticles to improve the BA observed after oral administration, and succeeded in preparing an orally disintegrating tablet containing FAM nanoparticles that can be redispersed after long-term storage and provide the enhancement of BA.

## 2 Materials and methods

### 2.1 Animals

All animal experiments were performed according to the guidelines of Kindai University and the Japanese Pharmacological Society. Six-week-old male Wistar rats (approximately 200 g) were provided by Kiwa Laboratory Animals Co., Ltd. (Wakayama, Japan) and were housed at 25°C. Water and a standard CE-2 diet (Clea Japan Inc., Tokyo, Japan) were freely provided. The experiments were approved on 1 April 2019 by Kindai University under the project identification code KAPS-31-014. In addition, the study was conducted in compliance with ARRIVE guidelines.

### 2.2 Chemicals

FAM powder, polyvinylpyrrolidone (PVP), gum arabic, isoflurane, D-mannitol, methyl p-hydroxybenzoate, and cytochalasin D were purchased from Wako Pure Chemical Industries Ltd. (Osaka, Japan). The Bio-Rad Protein Assay Kit and 2-hydroxypropyl-β-cyclodextrin (HPβCD) were obtained from Bio-Rad (CA, United States) and Nihon Shokuhin Kako Co., Ltd. (Tokyo, Japan), respectively. Nystatin was purchased from Sigma–Aldrich (St. Louis, MO, United States). Pentobarbital and 1-heptanesulfonic acid sodium salt were obtained from Tokyo Chemical Industry Co. Ltd. (Tokyo, Japan). Methylcellulose (MC) was purchased from Shin-Etsu Chemical Co. Ltd. (Tokyo, Japan). Rottlerin and dynasore were obtained from Nacalai Tesque (Kyoto, Japan). All chemicals used were of the highest commercially available purity.

### 2.3 Production of orally disintegrating tablets containing FAM micro- and nanoparticles

FAM powder, MC, and HPβCD were added to purified water (FAM-MP dispersions) and milled using a Bead Smash12 (Wakenyaku Co. Ltd., Kyoto, Japan) with 0.1 mm zirconia beads at 5,500 rpm for 30 s × 30 times at 4°C (Nagai et al., 2014; Nagai et al., 2019a; Nagai et al., 2019b; Deguchi et al., 2021; Otake et al., 2021; Nagai et al., 2022). The milled FAM dispersions were used as FAM-NP dispersions in this study. Subsequently, D-mannitol, PVP, and/or gum arabic were added to the FAM-MP and FAM-NP dispersions, and 0.7 mL of each FAM dispersion was set into a PTP sheet. The dispersions were then frozen for 24 h at −80°C. Afterwards, the dispersions were freeze-dried using the FREEZE DRYER FD-1000 (TOKYO RIKAKIKAI CO., LTD., Tokyo, Japan) for 48 h and used as an orally disintegrating tablet thereafter (FAM-MP and FAM-NP tablets). The condition of lyophilization (freeze-dry) were following: temperature −20°C, pressure 20 Pa, time 48 h. The compositions of FAM-MP and FAM-NP tablets are listed in Table 1. In this study, the FAM-MP and FAM-NP tablets were stored at standard conditions (12 h/day fluorescent light (07:00–19:00), 22°C, 30%–35% humidity) to evaluate for changes in stability of tablets.

**TABLE 1:** Compositions of FAM dispersions and tablets used in this study.

Formulation		Content (w/v%)						Treatment
		FAM	MC	HPβCD	Mannitol	PVP	Gum arabic	
Rp.1 (FAM-MP dispersions)		1	0.5	0.5				―
FAM-MP tablet		1	0.5	0.5	4	0.4	6	Freeze-drying
Rp.2 (FAM-NP dispersions)		1	0.5	0.5				Bead mill
FAM-NP tablet	Rp.3	1	0.5	0.5	4			Bead mill, Freeze-drying
	Rp.4	1	0.5	0.5		0.4		Bead mill, Freeze-drying
	Rp.5	1	0.5	0.5			6	Bead mill, Freeze-drying
	Rp.6	1	0.5	0.5	4	0.4	6	Bead mill, Freeze-drying
	Rp.7	1	0.5	0.5	4	0.4	10	Bead mill, Freeze-drying
	Rp.8	1	0.5	0.5	4	0.4	12	Bead mill, Freeze-drying
	Rp.9	1	0.5	0.5	4	0.4	16	Bead mill, Freeze-drying

### 2.4 Measurement of FAM concentration using high-performance liquid chromatography (HPLC)

FAM concentration was determined by HPLC using an LC-20AT HPLC system (Shimadzu Corp., Kyoto, Japan). A Luna^®^ 5 µm C18 column (100 Å, 150 × 4.6 mm, GL Science Co., Inc., Tokyo, Japan) was used for the measurement, and the mobile phase [one-heptanesulfonic acid sodium salt/acetonitrile/methanol (25/6/1)] was followed at 0.25 mL/min. The sample was mixed with an internal standard (methyl p-hydroxybenzoate) in methanol and 10 µL of the mixture was injected into the HPLC system using an SIL-20AC HPLC auto injector. In this study, the FAM levels were measured at 254 nm (wavelength for detection).

### 2.5 Dispersibility of FAM dispersions

The experiment was performed as described in our previous reports (Deguchi et al., 2021; Nagai et al., 2022). First, 3 mL of each FAM dispersion were incubated in 5 mL test tubes in the dark at 20°C for 28 days. Images of the FAM dispersions were captured with a digital camera. The dispersions (50 μL) were withdrawn from 5 mm under the surface at the indicated time intervals (0, 7, 14, 21, and 28 days), and the FAM concentration was measured using the HPLC methods described above. In this study, the difference in FAM concentrations before and after the start of the experiment was estimated, and the changes in FAM levels on the surface were defined as the dispersibility of the FAM dispersions.

### 2.6 Characterization of the FAM tablets and their redispersion

The characterization of the FAM tablets and their redispersion were evaluated in accordance with our previous study (Deguchi et al., 2021; Nagai et al., 2022). Briefly, the shape and dimensions of the tablets were analyzed using ImageJ image analysis software 1.53. Hardness was measured using a Force Tester MCT-2150 (A&D Co., Ltd., Toshimaku, Japan), and friability was measured using an Oriental Reaction Motor (ORIENTAL MOTOR Co., Ltd., Tokyo, Japan). The disintegration of FAM tablets was evaluated using NT-2H (TOYAMA SANGO Co. Ltd., Osaka, Japan). The friability and disintegration of the FAM tablets were measured according to the Japanese Pharmacopoeia (18th edition). The FAM tablets were dispersed in 10 mL of purified water and the redispersion, solubility, viscosity, and intestinal penetration were measured. The particle distributions of the FAM dispersions and redispersed FAM tablets were measured by Dynamic Light Scattering NanoSight LM10 (Quantum Design Japan, Tokyo, Japan), and the measurement time, wavelength, and viscosity were set to 60 s, 405 nm (blue) and 1.27 mPa s, respectively. Scanning electron microscopy (SEM) images were obtained using NeoScope™ JCM-7000 (JEOL Ltd., Tokyo, Japan). When the solubility was measured, the FAM nanoparticles and soluble FAM were separated by high centrifugation (1.0 × 10^5^ g) using a Beckman Optima™ MAX-XP Ultracentrifuge (Beckman Coulter, Osaka, Japan), and the FAM concentration in the supernatant was measured using the HPLC method described above. A powder X-ray diffraction (XRD) analyzer Mini Flex II (Rigaku Co., Tokyo, Japan) was used to evaluate the crystalline form of FAM in the tablet. The viscosity of the redispersed FAM tablet was measured at 20°C–40°C using an SV-1A viscometer (A&D Company, Limited, Tokyo, Japan).

### 2.7 Dissolution test of the FAM tablets

The FAM-MP and FAM-NP tablets (Rp.9 formulations shown in Table 1) were added to 0.5 mL of pH1.2 phosphate buffer and purified water in 1.5 mL tube at 37°C, and stirred at 50 rpm. After that, the dispersions were filtrated by 25 nm pore-size membrane filters (MF™-MEMBRANE FILTER, Merck Millipore, Tokyo, Japan) at the indicated time intervals (1, 2, 3, 4, and 5 min). The FAM concentration in the filtrate were measured using the HPLC methods described above. In this study, the pH1.2 phosphate buffer were prepared according to the dissolution test of Japanese Pharmacopoeia (18th edition).

### 2.8 *Ex-vivo* penetration of FAM tablet in isolated rat intestine

The *ex-vivo* intestinal penetration of the FAM-NP tablets was evaluated according to a previous study using methacrylate cells (Deguchi et al., 2021; Nagai et al., 2022). Seven-week-old Wistar rats were sacrificed by injecting a lethal dose of pentobarbital (200 mg/kg), and the jejunum was removed. The jejunum was placed on the methacrylate cell 37°C. The donor (apical) side in methacrylate cell were filled with 3 mL HEPES buffer [K_2_HPO_4_ (1 mM), glucose (5.5 mM), HEPES (10 mM), KCl (5.3 mM), NaCl (136.2 mM), and CaCl_2_ (1.7 mM), pH 7.4] containing redispersed FAM-NP tablet (13.8 μM), and other side [reservoir (basolateral) side] were filled with HEPES buffer. Next, samples (50 μL) were withdrawn from the reservoir side at the indicated time intervals (0.5, 1, 2, 3, 4, 5, and 6 h), and the FAM concentration and number of FAM nanoparticles in the collected samples were measured by HPLC and NanoSight LM10, respectively, as described above. Moreover, the *AUC*_0–6h_ in the reservoir chamber was estimated using the trapezoidal rule. The permeation area and dimensions of the intestinal tissue were determined as 0.3485 cm^2^ and 0.1 cm, respectively (n = 5).

In this study, the inhibition of energy-dependent endocytosis was performed under either cold conditions (4°C) (He et al., 2018) or in the presence of four pharmacological inhibitors of energy-dependent endocytosis. The relationships between energy-dependent endocytosis and intestinal penetration of FAM-NPs were investigated. Nystatin (54 μM) (Mäger et al., 2012), dynasore (40 μM) (Malomouzh et al., 2014), rottlerin (2 μM) (Hufnagel et al., 2009), and cytochalasin D (10 μM) (Mäger et al., 2012) were selected as inhibitors of caveolae-dependent endocytosis (CavME), clathrin-dependent endocytosis (CME), macropinocytosis (MP), and phagocytosis, respectively. These pharmacological inhibitors were dissolved in HEPES buffer containing 0.5% dimethyl sulfoxide (DMSO, vehicle), and the methacrylate cell-set intestine was treated starting 5 min before the experiment until the end of the experiment. The above experimental conditions were grouped into seven groups: normal condition (37°C, n = 9), cold condition (4°C, n = 6), vehicle (0.5% DMSO, n = 8) nystatin (n = 8), dynasore (n = 8), rottlerin (n = 8), and cytochalasin D (n = 8). Then, intestinal permeability was evaluated for each group. When intestinal tissue is destroyed, a rapid increase in drug penetration (burst) are observed. This study confirmed the viability of the intestinal tissue by confirming that these bursts do not occur.

### 2.9 Pharmacokinetic study of FAM tablet in the rat

Thirteen Wistar rats (7-week-old) were separated into two groups (6 and 7 rats), and the redispersed FAM-MP and FAM-NP tablets (0.8 mg/kg) were orally administered to rats fasted for 8 h (FAM-MP tablet, n = 6; FAM-NP tablet, n = 7). Blood (200 μL) was collected from the right jugular vein at the indicated time intervals (0, 10, 20, 30, and 360 min). The collected blood was centrifuged at 800 × g for 15 min at 4°C, and the supernatant was used for measurement. The FAM levels in the samples were measured using the HPLC methods described above, and the *AUC*_0–6h_ was estimated following the trapezoidal rule. The pharmacokinetics parameters were analyzed by Eqs 1, 2:

$$C_{FAM}=C_{0}\cdot e^{-k_{e}\cdot t}$$ (1)

$$C_{FAM}=\frac{k_{a}\cdot F\cdot D}{V_{d}\left(k_{a}-k_{e}\right)}\left(e^{-k_{e}\cdot t}-e^{-k_{a}\cdot t}\right)$$ (2)

The distribution volume (*V*_d_) and elimination rate constant (*k*_e_) were calculated using Eq. 1 and data (0, 0.25, 0.5, 1, 2, 3, 6, and 24 h, *t*) after a single injection (0.3 mL) of FAM solution in DMSO (0.04 mg/kg) into the femoral vein, and levels of 64.0 mL and 7.8 × 10^−2^ h^−1^, respectively, were obtained. The apparent absorption rate constant (*k*_a_) was estimated according to Eq. 2. The *D* and *F* show dose (0.8 mg/kg), and fraction of FAM absorbed, respectively.

### 2.10 Statistical analysis

Statistical analysis was performed using the JMP ver. 5.1 (SAS Institute). Student’s t-test and one-way analysis of variance (ANOVA) followed by Dunnett’s multiple comparisons were used for statistical analysis. The sample numbers (n) are shown in Fig. legends, and data are expressed as mean ± standard error (S.E.) of the mean.

## 3 Results

### 3.1 Preparation of dispersions containing FAM nanoparticles by bead-milling treatment

**FIGURE 1:**
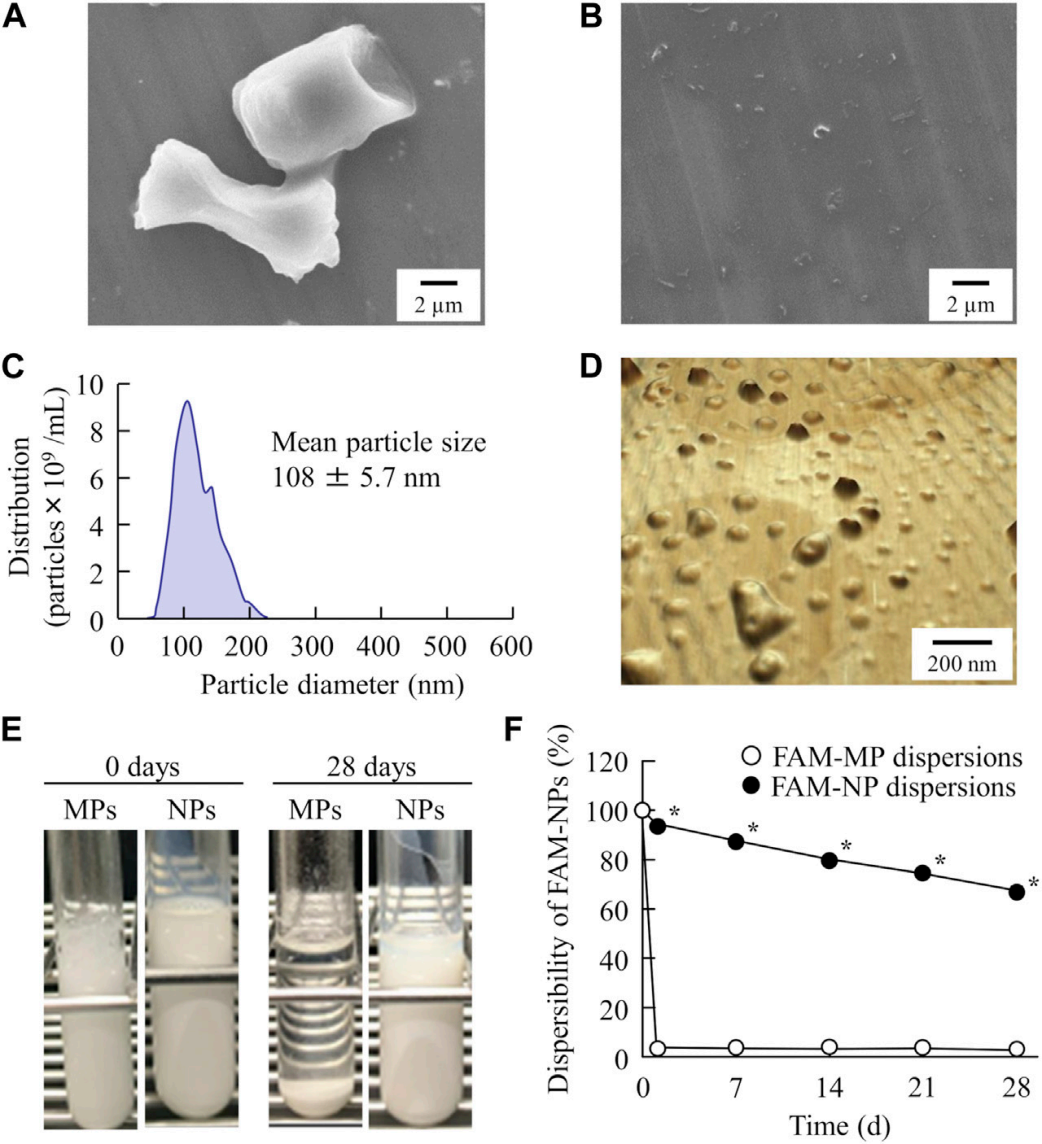
Evaluation of FAM particles in the FAM-MP and FAM-NP dispersions. SEM image of FAM in FAM-MP **(A)** and FAM-NP **(B)** dispersions. Particle size frequencies **(C)** and atomic force micrograph **(D)** of FAM in FAM-NP dispersions. Photograph of FAM-MP and FAM-NP dispersions immediately (0 days) and 28 days after preparation **(E)**, and dispersibility of FAM in FAM-MP and FAM-NP dispersions for 28 days **(F)**. n = 10. *p < 0.05 vs. FAM-MP dispersions. The FAM nanoparticles were produced by bead-milling treatment, and the particle size was approximately 50–220 nm. The sedimentation in the FAM-NP dispersions was gradual in comparison with that in the FAM-MP dispersions.

First, we prepared FAM nanoparticle dispersions using the breakdown method to design an orally disintegrating tablet containing FAM nanoparticles (FAM-NP tablet). Figures 1A–D show the particle size distribution of FAM with and without bead-milling treatment. The bead-milling treatment decreased the particle size of FAM to the nanoscale. Specifically, the mean particle size of FAM was 23.7 ± 2.1 µm and 108 ± 5.7 nm without and with bead-milling treatment, respectively. Second, we measured the effect of the bead-milling treatment on stability of FAM particles (Figures 1E, F). Without bead-milling treatment, the FAM was precipitated 24 h after preparation; however, the dispersibility of FAM was increased and the sedimentation time was prolonged by the bead-milling treatment.

### 3.2 Design of orally disintegrating tablets containing FAM nanoparticles

**FIGURE 2:**
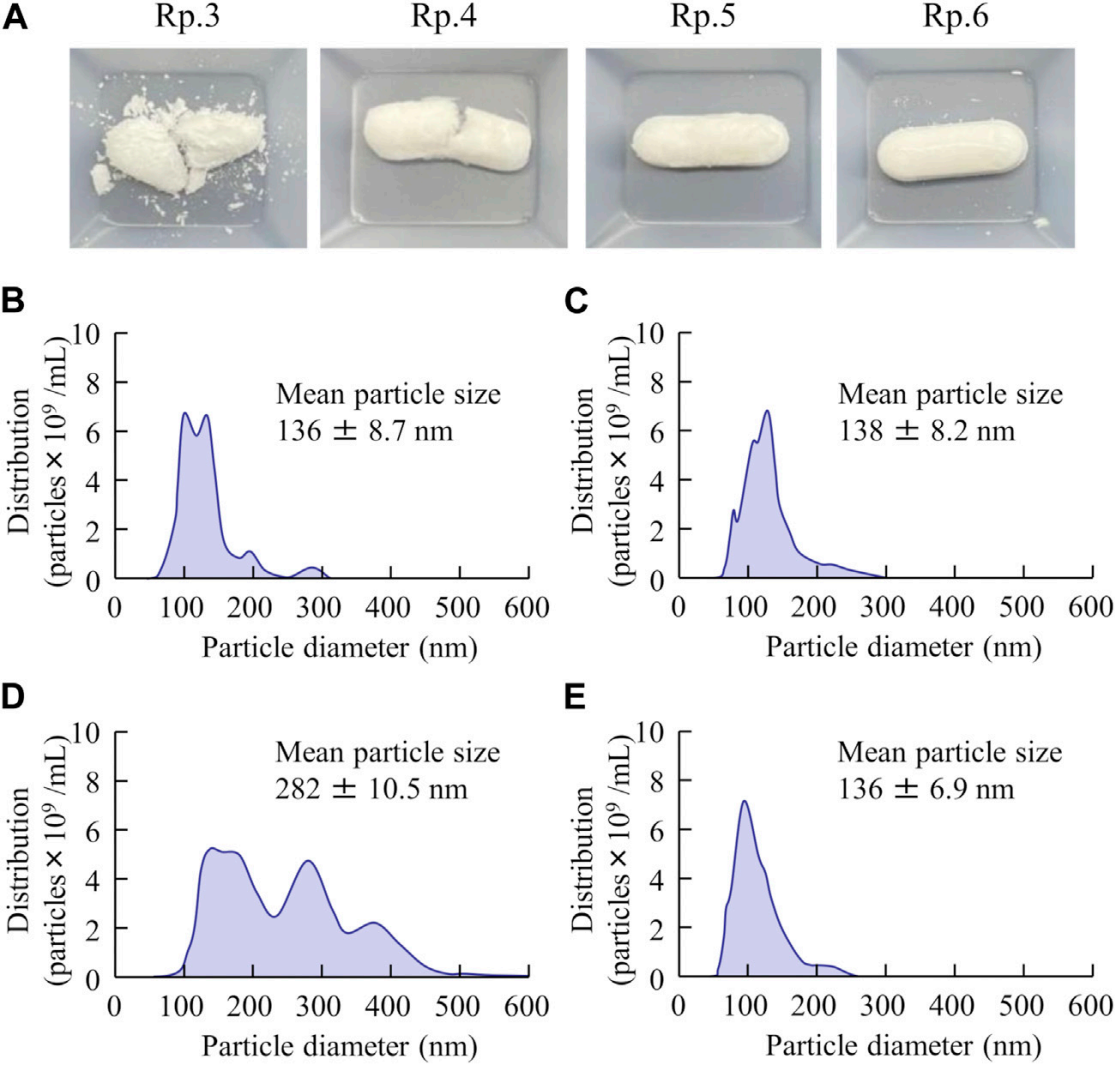
The image and particle size frequencies of FAM-NP tablets are listed in Table 1. Photograph of FAM tablets **(A)**, (Rp. 3–6 formulations). Particle size frequencies of FAM in redispersed Rp.3 **(B)**, Rp.4 **(C)**, Rp.5 **(D)**, and Rp.6 **(E)** formulations. The addition of D-mannitol, PVP, and gum arabic formed a tablet containing FAM nanoparticles, and the FAM particles in the redispersion of the FAM-NP tablet (Rp.6 formulations) were nano-sized.

**FIGURE 3:**
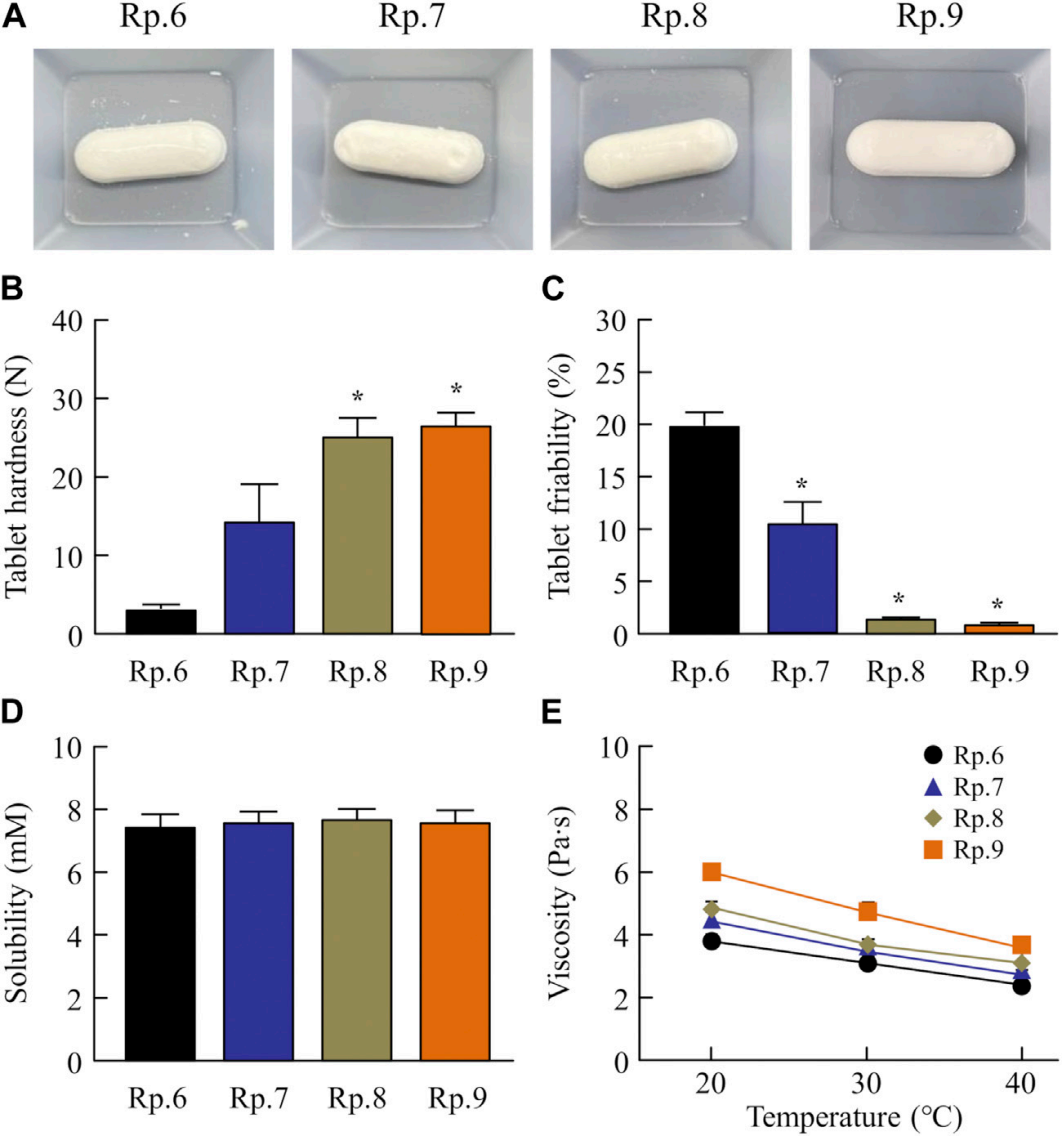
Effect of gum arabic contents on the hardness, friability, solubility, and viscosity of the FM-NP tablets shown in Table 1. Photograph **(A)**, hardness **(B)**, and friability **(C)** of the FAM-NP tablets (Rp.6–9 formulations). Changes in FAM solubility **(D)** and viscosity **(E)** of redispersed FAM tablet (Rp.6–9 formulations) at 20°C–40°C. n = 10. *p < 0.05 vs. Rp.6 formulations for each category. The gum arabic contents enhanced the hardness and viscosity and decreased the friability of the FAM-NP tablets. FAM solubility was similar among the Rp. 6–Rp.9 formulations.

Next, we prepared an orally disintegrating tablet containing FAM nanoparticles using the FAM-NP dispersions prepared above. Figure 2A shows an image of the FAM-NP tablets, and Figures 2B–E show the FAM particle distribution in the redispersion of the FAM tablet. Although the FAM particles in the redispersion of freeze-dried FAM-NP tablets with D-mannitol (Rp.3 formulations) or PVP (Rp.4 formulations) were nano-sized, the Rp.3 and Rp.4 formulations easily disintegrated and could not maintain their shape as tablets. In contrast, the freeze-dried FAM-NP tablet with gum arabic (Rp.5 formulations) formed and remained a tablet. However, upon redispersion, the particles in the Rp.5 formulations aggregated (282 ± 10.5 nm). When combined, the freeze-dried FAM-NP tablet with D-mannitol, PVP, and gum arabic maintained its shape as a tablet (Rp.6 formulations), and upon redispersion, the mean particle size of FAM in the FAM-NP tablets was 136 ± 6.9 nm. Furthermore, we demonstrated the relationship between characterization as a tablet and nanoparticulation in redispersion (Figure 3). Both the hardness and friability of the FAM-NP tablet increased with the gum arabic content. While the solubility of FAM in the redispersed FAM-NP tablet did not change, viscosity was enhanced by the addition of gum arabic. Indeed, the viscosity of Rp.9 formulations was 1.59-fold higher than that of the Rp.6 formulations at 20°C.

### 3.3 Stability and dissolution pattern of orally disintegrating tablets containing FAM nanoparticles

**FIGURE 4:**
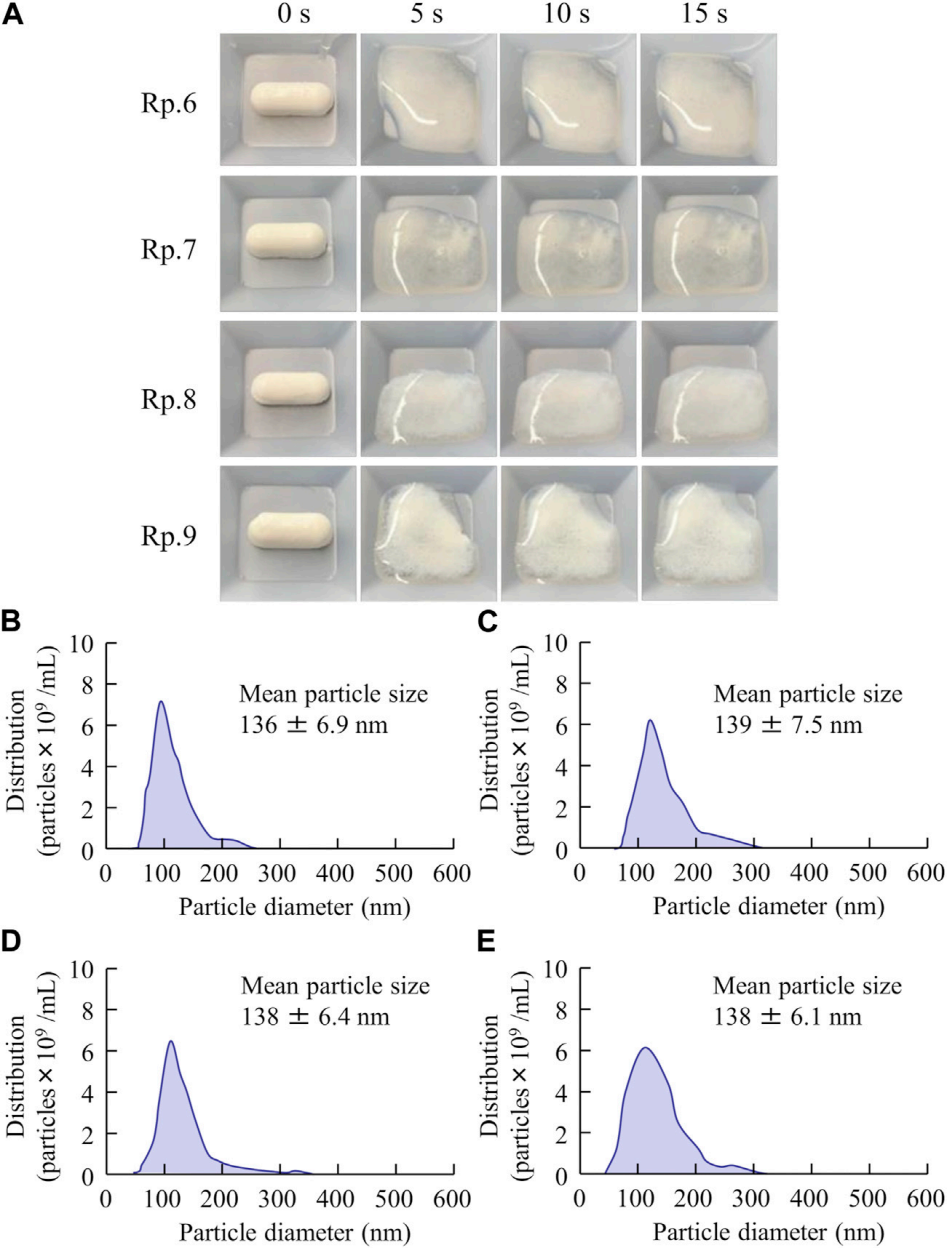
Effect of gum arabic contents on the disaggregation time and particle distribution of the FAM-NP tablets shown in Table 1. Photograph in the disaggregation process of the FAM-NP tablets **(A)**, (Rp. 6–9 formulations). Particle distribution of the redispersed Rp.6 **(B)**, Rp.7 **(C)**, Rp.8 **(D)**, and Rp.9 **(E)** formulations. The Rp.6–Rp.9 formulations were disaggregated within 15 s after addition to purified water. The FAM particles in the redispersed FAM-NP tablet were nano-sized.

Figure 4 shows the disaggregation time and particle distribution when FAM-NP tablets were added to water. The disaggregation times of the Rp.6–Rp.9 formulations were similar, with all being disaggregated in less than 15 s. In addition, the FAM particles in the redispersion of the FAM-NP tablet were nano-sized, regardless of the gum arabic content. The mean particle size of FAM in the redispersion of the FAM-NP tablet (Rp.9 formulations) was 138 ± 6.1 nm. We also determined the powder X-ray diffraction patterns of the Rp.9 formulations (Supplementary Figure S1). Some peaks of crystalline FAM were detected in the FAM-NP tablet; however, the peaks were different between FAM treated with freeze-drying and bead-milling. In addition, the behavior of the XRD pattern was close to amorphous. Therefore, the crystal forms of FAM in the FAM-NP tablet may mix crystal polymorphs and amorphous phases. In this study, we also measured the tablet form of the Rp.9 formulations. The major axis, minor axis, thickness, and weight of tablet were 11.85 ± 0.01, 7.07 ± 0.01, 6.79 ± 0.01 mm, 149.9 ± 0.9 mg, respectively (n = 30). Table 2 shows the physical properties of the redispersed Rp.9 formulations after 3 months of storage as tablets. The 3-month-stored FAM-NP tablet disintegrated in 3.5 s, and the particle size of FAM in the redispersed Rp.9 formulations was 141 nm (Supplementary Figure S2). In addition, there were no difference in the hardness, friability, solubility, and viscosity of the immediately prepared and 3-month-stored formulations (Table 2). Table 3 shows the dissolution test of the FAM tablets. The solubility of FAM in pH1.2 buffer were higher than that in purified water, and the dissolution rates of FAM in FAM-NP tablet was significantly increased in comparison with FAM-MP tablet in the both pH1.2 buffer and purified water (Table 3).

**TABLE 2:** Characteristics of the FAM-NP tablet after 3 months of storage.

Formulation	Mean particle size (nm)	Disaggregation time (s)	Hardness (N)	Friability (%)	Solubility (mM)	Viscosity (Pa∙s, 30°C)
FAM-NP tablet	141 ± 6.6	3.5 ± 0.5	26.2 ± 4.3	0.79 ± 0.1	7.84 ± 0.6	4.8 ± 0.5

The formulations shown in Table 1 were used in this study (n = 8).

**TABLE 3:** The FAM-MP, and FAM-NP tablets (Rp.9 formulation) shown in Table 1 were used in this study (n = 3). *p < 0.05 vs. FAM-MP tablet for each category.

	pH1.2 buffer (mM)		Purified water (mM)	
	FAM-MP tablet	FAM-NP tablet	FAM-MP tablet	FAM-NP tablet
1 min	13 ± 1.3	32 ± 4.5*	1.0 ± 0.4	3.7 ± 0.9*
2 min	25 ± 1.3	51 ± 3.9*	1.9 ± 0.6	5.3 ± 0.7*
3 min	33 ± 1.2	65 ± 3.6*	2.7 ± 0.6	6.8 ± 0.5*
4 min	39 ± 1.1	71 ± 2.4*	3.5 ± 0.8	7.3 ± 0.4*
5 min	43 ± 1.1	73 ± 2.3*	4.1 ± 0.5	7.5 ± 0.2*

### 3.4 Transintestinal pathway of FAM in the FAM-NP tablet

**FIGURE 5:**
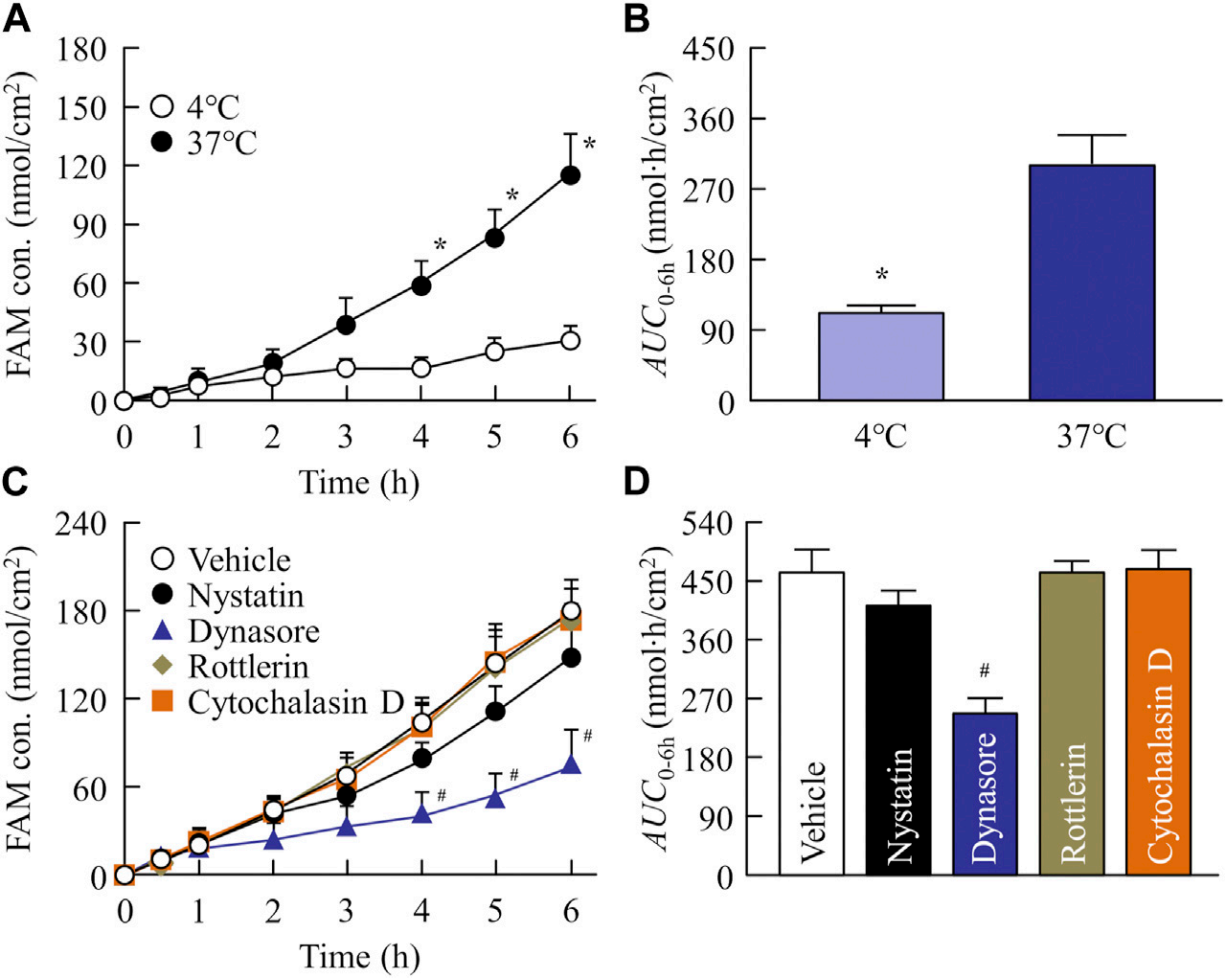
Evaluation of transintestinal penetration of drug using rat intestines co-treated with FAM-NP tablet (Rp.9 formulations) and endocytosis inhibitors. Penetration profile **(A)** and *AUC*_0–6h_ **(B)** of FAM in the FAM-NP tablet under cold (4°C) and normal (37°C) conditions. Penetration profile **(C)** and *AUC*_0–6h_ **(D)** of FAM in the rat intestines co-treated with FAM-NP tablet and endocytosis inhibitors [vehicle (0.5% DMSO), nystatin, dynasore, rottlerin, cytochalasin D]. n = 6–9. *p < 0.05°C vs. 37°C for each category. ^#^ *p* < 0.05 vs. the vehicle for each category. The intestinal penetration of the FAM-NP tablet was attenuated by the inhibitor of clathrin-mediated endocytosis, dynasore.

Our previous reports showed that energy-dependent endocytosis is involved in high intestinal penetration of drug nanoparticles (Deguchi et al., 2021; Nagai et al., 2022). Based on these previous studies, we investigated the relationship between intestinal penetration and energy-dependent endocytosis using low temperature (4°C) and phagocytosis inhibitors (nystatin, dynasore, rottlerin, and cytochalasin D). Figures 5A, B show the changes in the penetration profile (A) and area under the drug concentration–time curve (*AUC*_0–6h_, B) of the redispersed FAM-NP tablet at 4°C and 37°C. Intestinal penetration in the FAM-NP tablet was attenuated under cold conditions, and the *AUC*_0–6h_ under cold conditions was 37.3% of the FAM-NP tablet at 37°C. Figures 5C, D show the changes in the penetration profile (C) and *AUC*_0–6h_ (D) in the redispersed FAM-NP tablet treated with pharmacological inhibitors of energy-dependent endocytosis. Although treatment with rottlerin and cytochalasin D did not affect the intestinal penetration of the FAM-NP tablet, it tended to decrease when treated with nystatin. In addition, the intestinal penetration in the FAM-NP tablet was significantly decreased by treatment with dynasore, and the *AUC*_0–6h_ of the FAM-NP tablet when treated with dynasore was 53.2% of that of the FAM-NP tablet when treated with vehicle. We also measured the number of FAM nanoparticles transferred from the donor side (apical) to the reservoir side (basolateral). No FAM nanoparticles were detected on the basolateral side in either the group with or without pharmacological inhibitor treatment, and the FAM transferred to the basolateral side was only dissolved-FAM (FAM solution).

### 3.5 Absorption of FAM from the FAM-MP and FAM-NP tablets

**FIGURE 6:**
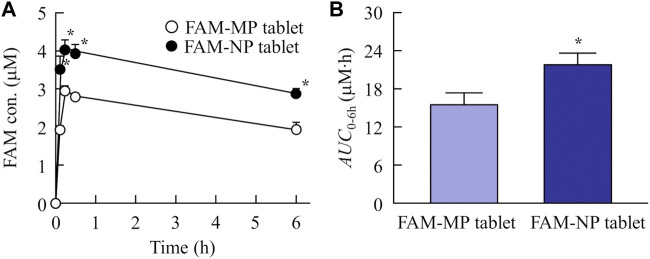
Changes in plasma FAM concentration in the rats orally administered the FAM-MP and FAM-NP tablets (Rp.9 formulations). The absorption profile **(A)** and *AUC*_0–6h_ **(B)** of FAM in the rats orally administered the FAM-MP and FAM-NP tablets (Rp.9 formulations). n = 6–7. *p < 0.05 vs. FAM-MP tablet for each category. The plasma FAM levels in the rats administered the FAM-NP tablet was significantly higher than that in the rats administered the FAM-MP tablet.

Figure 6 shows the plasma FAM concentration in rats orally administered FAM-MP and FAM-NP tablets, and Table 4 summarizes the pharmacokinetic parameters calculated from the *in vivo* intestinal penetration data. The *T*_max_ of the FAM-NP tablet was shortened in comparison with FAM-MP tablet, and *k*_a_, and *C*_max_ of the FAM-NP tablet were significantly higher than those of the FAM-MP tablet. Moreover, the *AUC*_0–6h_ of the FAM-NP tablet was 1.41-fold of the FAM-MP tablet.

**TABLE 4:** The FAM-MP and FAM-NP tables (Rp.9 formulation) shown in Table 1 were used in this study (n = 3). The ke was 7.8 × 10^−2^ h^−1^. n = 6–7. *p < 0.05 vs. FAM-MP tablet for each category.

Formulation	*k*_a_ (h^-1^)	*T*_max_ (h)	*C*_max_ (µM)
FAM-MP tablet	10.3 ± 0.9	0.48 ± 0.03	3.01 ± 0.25
FAM-NP tablet	18.8 ± 1.3*	0.29 ± 0.02*	4.13 ± 0.40*

## 4 Discussion

Drug dispersions containing solid nanoparticles act as a drug delivery system to enhance the permeability of BCS class III drugs, increasing their BA. However, dispersions containing solid nanoparticles are prone to chemical and physical instability because they possess a high Gibbs free energy owing to their very large, exposed surface areas (Kumar and Burgess, 2012). In this study, we attempted to overcome these issues associated with oral formulations of BCS class III drugs by designing molded tablets containing FAM nanoparticles to improve the low oral BA.

First, we attempted to prepare an orally disintegrating tablet containing the FAM nanoparticles. The molded tablets are commonly used in tablet making, however, the molding process hardens drug particles, making them difficult to redispersible as nanoparticles. Therefore, we applied the method using the combination of bead milling and freeze-drying treatment to produce the orally disintegrating tablet in this study. Breakdown methods are widely used to prepare nanocrystalline dispersions (Kumar and Burgess, 2014), and determining the content and type of additives is important for producing good quality nanoparticles (Nagai et al., 2014). In this study, we selected MC and HPβCD as additives in the bead milling treatment and found that MC enhanced the crushing efficiency in the bead mill while HPβCD prevented the aggregation of drug particles (Nagai et al., 2014; Nagai et al., 2019a; Nagai et al., 2019b; Deguchi et al., 2021; Otake et al., 2021; Nagai et al., 2022). Furthermore, excipients, dispersing agents, and binders are necessary to produce orally disintegrating tablets. We chose mannitol, PVP, and gum arabic for each of these, respectively. Freeze-drying treatment was used to solidify the nanocrystalline dispersions in accordance with a previous study (Nagai et al., 2022). The particle size of FAM was approximately 50–220 nm after the bead-milling treatment (Figure 1), and an orally disintegrating tablet that retained FAM nanoparticles even when redispersed was successfully prepared by the addition of these additives and freeze-drying treatment (Figure 2). In addition, gum arabic increased the hardness and viscosity and decreased the friability of the tablet containing FAM nanoparticles, and these tablets were disaggregated in less than 15 s (Figures 3, 4). Based on these results, Rp.9 formulations were used in subsequent experiments because they were the hardest of the formulations and maintained good disintegration and redispersion in this study.

Next, we investigated whether orally disintegrating tablets containing nanoparticles can be provided after long-term storage. The physical characteristics (hardness, friability, solubility, and viscosity) of the 3-month-stored FAM-NP tablet were similar to those of the immediately prepared tablet (Table 2) and quickly disintegrated in 3.5 s. Furthermore, the particle size of FAM in the redispersion was nano-sized even after 3 months of storage (Table 2; Supplementary Figure S2). These results suggest that the chemical and physical instabilities of dispersions containing solid nanoparticles may be improved by preparing orally disintegrating tablets using freeze-drying treatment and the additives used in this study.

Additionally, it is important to demonstrate whether orally disintegrating tablets containing nanoparticles of BCS class III drugs provide high oral BA. Therefore, we compared the plasma FAM levels of rats administered FAM-MP and FAM-NP tablets (Table 4; Figure 6). The plasma FAM levels and *k*_a_ in rats administered the FAM-NP tablet were higher than those in rats administered the FAM-MP tablet. A mixture of the crystal polymorph and amorphous phase was observed in the FAM-NP tablet (Supplementary Figure S1), and the amorphous phase increases the solubility and dissolution rate, resulting in the enhancement of intestinal penetration (Schittny et al., 2020). Moreover, the Ostwald-Freundlich equation predicts that the solubility of solid nanoparticles is higher than that of solid microparticles. The solubility of FAM in the FAM-NP tablet was 1.55-fold higher than that of the FAM-MP tablet (4.9 ± 0.5 mM, n = 6). Furthermore, we showed that the dissolution rate in the FAM-NP tablet was significantly higher than that in FAM-MP tablet in the both pH1.2 buffer and purified water (Table 3). These enhanced solubility and dissolution rates may lead to an improvement in the inherent low BA of BCS class III drugs (Table 3; Figure 3). In addition to the increase in both solubility and dissolution rate, cell uptake by energy-dependent endocytosis also improves low intestinal penetration (Deguchi et al., 2021; Nagai et al., 2022). Therefore, we investigated the relationship between BA after oral administration and energy-dependent endocytosis in the FAM-NP tablet. Previous studies have reported that energy-dependent endocytosis was inhibited under low-temperature conditions (4°C) (He et al., 2018). Similarly, in this study, the intestinal penetration of the FAM-NP table decreased at lower temperatures, with an *AUC*_0–6h_ at 37°C 2.68-fold that of the FAM-NP tablet at 4°C (Figures 5A, B). Furthermore, we used pharmacological inhibitors of energy-dependent endocytosis to investigate the involvement of endocytosis in the intestinal penetration pathway of the FAM nanoparticles. Of the four inhibitors used in this study (nystatin, dynasore, rottlerin, and cytochalasin D), only the inhibitor of clathrin-mediated endocytosis (dynasore) significantly attenuated the intestinal penetration of FAM-NP tablet (Figures 5C, D). As a result, clathrin-mediated endocytosis potentially improves the oral BA of FAM, a BCS class III drug, from the FAM-NP tablet as well as enhances its solubility. In this study, we demonstrated whether FAM nanoparticles could pass intact after being taken up into the small intestine by clathrin-mediated endocytosis. In the *ex-vivo* penetration experiment of FAM tablets using isolated rat intestines, no FAM nanoparticles were detected on the basolateral side, only dissolved-FAM (FAM solution). This result suggests that FAM-NPs dissolved when they penetrated the small intestine. Further studies are needed to elucidate the detailed mechanism of transintestinal penetration *via* clathrin-mediated endocytosis of FAM nanoparticles. Moreover, it is important to clarify the effects of oral administration of FAM-NP tablets. Therefore, we are planning to evaluate the therapeutic effect of FAM-NP tablets on gastric and duodenal ulcers using animal models.

## 5 Conclusion

**FIGURE 7:**
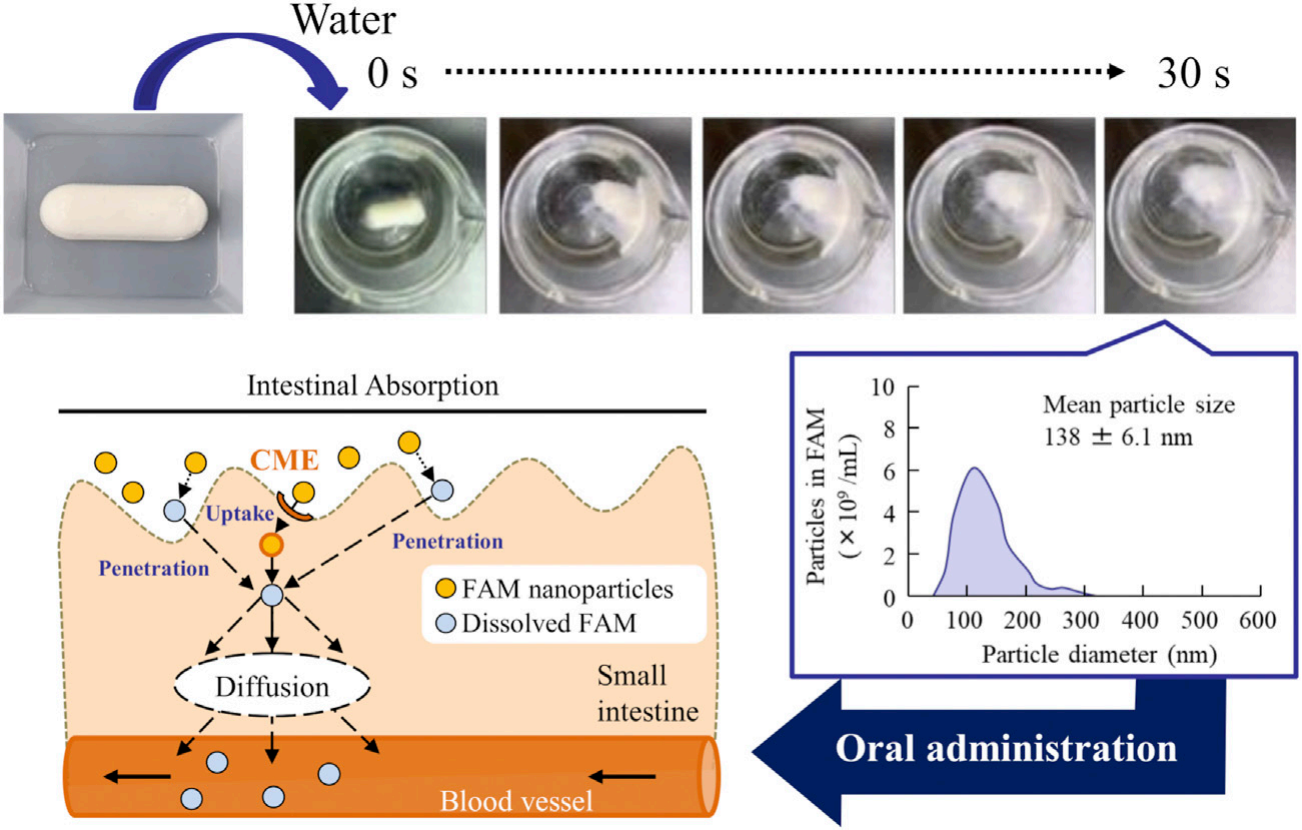
Mechanism for intestinal absorption after the orally administration of FAM-NP table.

We succeeded in preparing an orally disintegrating tablet containing FAM-NPs that can be redispersed after long-term storage. In addition, these formulations improved the low mucosal permeability and intestinal absorption of FAM and overcame the issues associated with oral formulations of BCS class III drugs (Figure 7). These findings provide significant information for the design of nanomedicines for increasing oral BA.

## Data Availability

The original contributions presented in the study are included in the article/Supplementary Material, further inquiries can be directed to the corresponding author.
