# Supplementary figures and images for: Orally disintegrating tablets containing famotidine nanoparticles provide high intestinal absorbability via the energy-dependent endocytosis pathway
Source: Front Bioeng Biotechnol. 2023 Mar 10;11:1167291. doi: 10.3389/fbioe.2023.1167291 (PMC10036753; doi:10.3389/fbioe.2023.1167291)

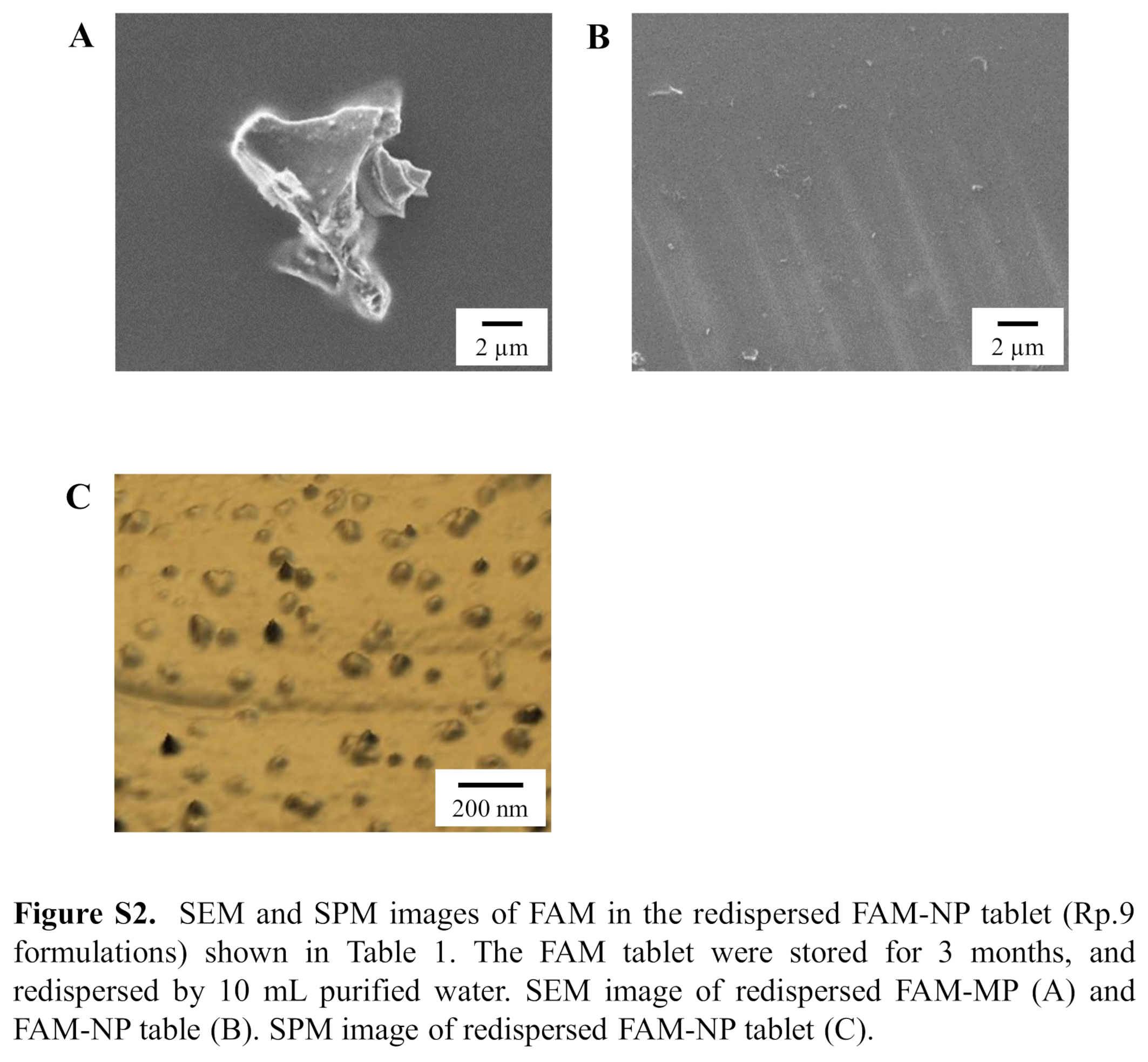

Supplement: Supplementary file 1 [file Image2.TIF]

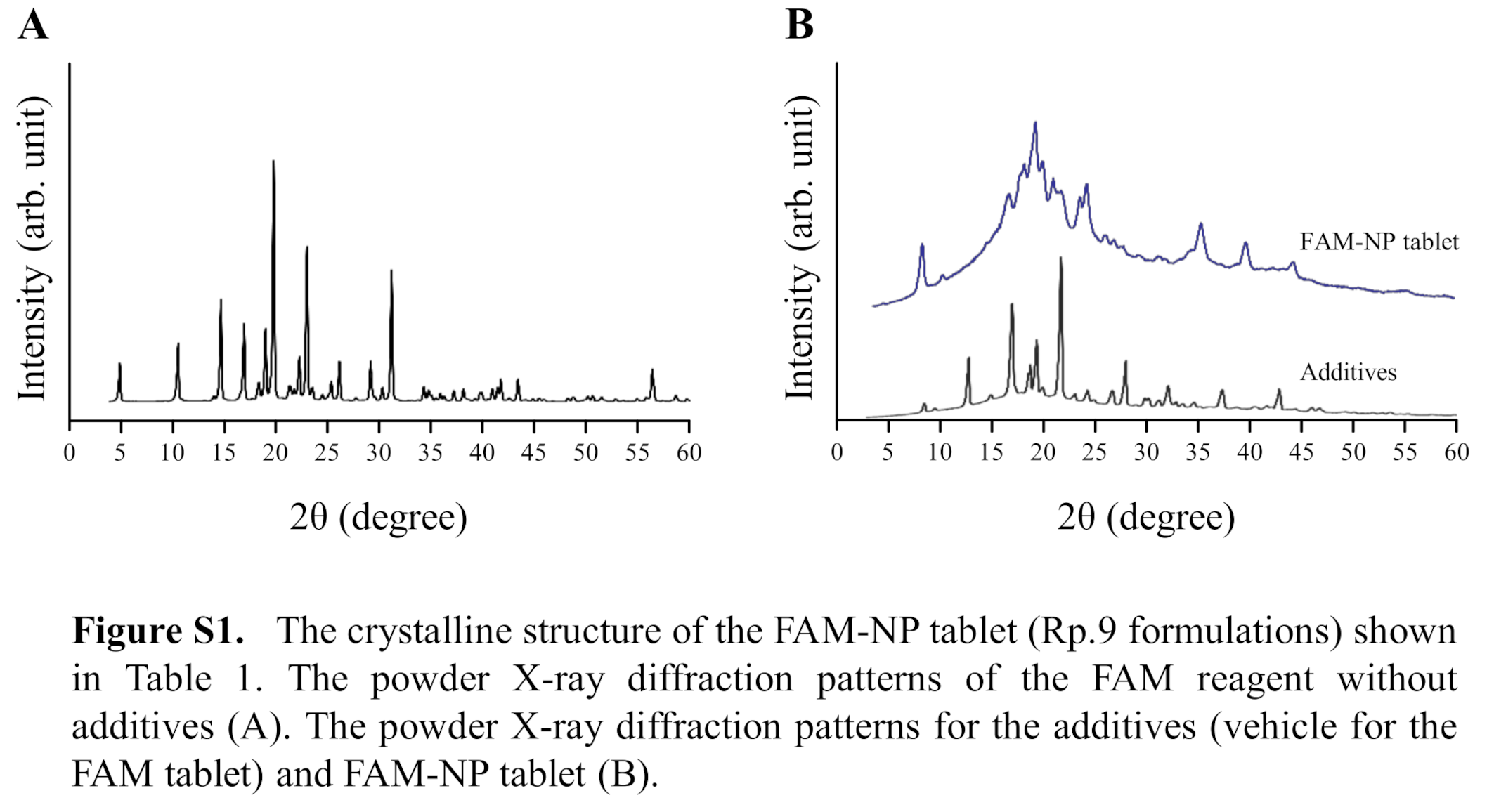

Supplement: Supplementary file 2 [file Image1.TIF]
